# Dialyzed Iron in the Treatment of Arsenical Poisoning

**Published:** 1882-08

**Authors:** 


### Dialyzed Iron in the Treatment of Arsenical Poisoning.

Dr. Bullard, in a communication to the *Medical and Surgical Reporter*, gives his experience, as follows: In the smelting of lead and silver ores, one of the worst features is the constant inhalation of arsenical fumes. When first employed by the Alta Montana Company, to take charge of their hospital, a number of cases of arsenical poisoning came under my observation, and they were the more difficult to treat on account of their complication with "leading." I tried the various remedies recommended for such cases, with but poor results. At times I felt that old saying, "Throw physic to the dogs," was but too true and applicable. At last I was led to try dialyzed iron, and was met in all cases with most gratifying success, as is evidenced by the following cases:

Two carpenters were engaged in roofing a portion of the smelting building, and were in such a position that the wind carried the fumes into their faces. Some workmen below noticed one of the men swaying to and fro, and about ready to fall, while the other was laboring hard to reach the ground. They were helped to the hospital, and were suffering with severe pain in stomach and bowels, nausea, vomiting, vertigo, and with a profuse "nose-bleed," tremor in lower limbs, and almost prostration. A wineglassful of dialyzed iron was given immediately. The nausea ceased, and at the end of one hour the men were able to walk to their cabins, carrying with them a bottle of the iron, to be taken in drachm doses every half hour. At the end of twenty-four hours they complained only of weakness, such as would result from a severe diarrhœa. The second day they resumed work, entirely free from all pain and effects of the arsenic. A number of men employed about the smelting furnace, and especially in dipping the molten lead, have been apparently prostrated, from the effects of the fumes, and were in every case relieved by dialyzed iron. A mild purgative was given within twelve hours. I have recommended, and, indeed, insisted on, every man who is exposed to the arsenical fumes, taking a dose of the iron daily. The consequence has been, that we have had but one case of poisoning needing hospital treatment, and this one insisted that his case was one of "indigestion and dyspepsia," and would take nothing till compelled to enter the hospital, where, under the administration of dialyzed iron, he speedily recovered.

In the past two years, I think I am safe in saying that fully two hundred cases of arsenical poisoning have been cured in this camp by dialyzed iron. I could cite any or all of them, with symptoms, treatment etc., but I think it unnecessary, as they so nearly resemble those already mentioned; suffice it to say, that all experienced the nausea, griping, vomiting, muscular tremor, etc. I have given the iron, in half-ounce doses, three times daily, with no constitutional disturbances whatever, even after ten and often twenty days' administration. The teeth are not discolored, bowels not constipated, and digestion not deranged.

The men have learned its virtues, and come regularly with "please fill my iron bottle again." They will not do without it, any more than an Irishman will without his "salts and senna." It has saved many a man his wages and many a day of sickness. In fact, I feel convinced that this preparation is indispensable where men are liable to inhale the fumes of arsenic.

Without a remedy of this kind, I am satisfied no man, however strong, could inhale the fumes incident to smelting, where the ores contain arsenic, and stand it more than three or four days. I can fully and confidently recommend this preparation of iron to the profession, and even to foremen of smelting works where there is no physician, for it is harmless and invaluable. "An ounce of prevention is worth a pound of cure;" or a "pound of cure," is worth infinitely more to a company, than are hospitals full of men poisoned with arsenic. Our hospital has been built, medicines bought, physicians and nurses paid, and accommodation for thirty beds provided, inside of two years, by a small monthly assessment on each miner and laborer employed by the company, and all are satisfied; none more so than the smelter hands, who can and do get a "bottle of that iron," and keep at work.

The preparation which I have used, and to the good effects of which I can testify, is Wyeth's, of Philadelphia.

